# Case studies in physiology: Nocturnal cardiorespiratory adaptive differences between an Italian trekker and a Nepali guide

**DOI:** 10.14814/phy2.14537

**Published:** 2020-08-18

**Authors:** Danilo Bondi, Suwas Bhandari, Vittore Verratti

**Affiliations:** ^1^ Department of Neuroscience, Imaging and Clinical Sciences University “G. d’Annunzio” of Chieti – Pescara Chieti Italy; ^2^ Wenzhou Medical University Wenzhou China; ^3^ Department of Psychological, Health and Territorial Sciences University “G. d’Annunzio” of Chieti – Pescara Chieti Italy

**Keywords:** blood pressure, breathing, Himalayas, hypobaric hypoxia, physiological monitoring, sleep

## Abstract

The cardiopulmonary system is a physiological cornerstone in the adaptive response to hypobaric hypoxia. Portable devices make it feasible nowadays to precisely assess the response to high altitude (HA) expeditions. In this study, we investigated breathing and arterial blood pressure responses during a Himalayan trek from 665 m to 4,780 m altitude in a white European (Italian) sojourner and a native Nepali (Tamang) guide, both healthy males. Resting diurnal and nocturnal data were acquired by means of ambulatory blood pressure monitoring (ABPM) and sleep apnea monitoring. We found an increase in the mean diurnal arterial blood pressure. Nocturnal blood pressure dipping was confirmed at all altitudes. Oxygen saturation decreased at altitude, with its additional nocturnal fall. Sleep apneic episodes, present in the Italian only, increased with altitude. We conclude that the nocturnal, more than diurnal, cardiorespiratory function is affected by HA hypoxia. Further studies should address the role of ethnicity, medications, and sociodemographic factors in the cardiorespiratory responses to hypobaric hypoxia.

## Graphical abstract

In this study, we investigated cardiopulmonary nocturnal and diurnal responses during a Himalayan trek in a white European (Italian) sojourner and a native Nepali (Tamang) guide, by means of ambulatory blood pressure and sleep‐apnea monitoring. We found an increase in the mean BP. Oxygen saturation decreased at altitude, with its additional nocturnal fall. Sleep apneic episodes increased with altitude.

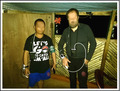

## 1 INTRODUCTION

High altitude (HA) has a multitude of pathophysiological effects (Young & Reeves, 2002). The condition is characterized by hypobaric hypoxia, decreased air density, and cold stress. Inspired oxygen pressure (PO_2_) and, consequently, arterial and tissue PO_2_ decrease. Nevertheless, there are native populations living at HA, such as Sherpas in the Himalayas, Tibetans on the Tibetan Plateau, or Andeans in the Andes. These peoples are chronically adapted to chronic HA in all body systems (Horscroft et al., 2017; Julian & Moore, 2019). Concerning cardiorespiratory function, adaptive responses to HA consist of increases in cardiac output and ventilation, and increases in the number of capillaries and mitochondria (Di Giulio et al., 2003; Mohsenin, 2015). In non‐acclimatized lowlanders, HA hypoxia causes an increase in cardiac output in a few days, primarily explained by increased heart rate (HR). In contradistinction, in HA‐acclimated lowlanders as well as in natives, cardiac output stays similar to that at sea level (Naeije, 2010). With acute ascent, HR at rest and at a standardized workload is higher than that at sea level, resulting in a systolic volume reduction (Princi, Zupet, Finderle, & Accardo, 2008).

Acute exposure to HA also increases blood pressure (BP), especially in the first days of exposure in lowlander (Calbet & Lundby, 2009) which persists after acclimatization (Parati et al., 2015) and is accompanied by reduced nocturnal BP dipping, due likely to a nighttime reduction in PO_2_ (Parati et al., 2014). The chronic response is an increase in BP, despite improved systemic O_2_ delivery with acclimatization (Calbet, 2003). The association of hypertension with altitude is population specific, for example Andean residents have a low prevalence of hypertension, whereas Tibetan highlanders have a high prevalence (Norboo et al., 2015). HA also evokes bradyarrhythmia during apneic periods in lowlanders, but not in Sherpas (Busch et al., 2018). However, population specificity of BP response to middle‐term hypobaric hypoxia remains to be elucidated.

HA critically affects respiration. A hypobaric reduction in alveolar PO_2_ limits the perimetric oxygen supply leading to a drop in arterial oxygen saturation (SaO_2_) (Calbet & Lundby, 2009). A hallmark of HA is periodic breathing, especially during non‐REM sleep (Ainslie, Lucas, & Burgess, 2013). Periodic breathing is characterized by periods of apnea lasting for 10–15 s, followed by hyperpnea caused by increased central chemoreceptor sensitivity to PaCO_2_ accumulation (Mohsenin, 2015). The hypoxic ventilatory response (HVR) to acute hypoxia is increased at HA, facilitating and extending nighttime periodic breathing (Lahiri, Maret, Sherpa, & Peters, 1984). Periodic breathing, duration and efficiency of sleep, and nighttime desaturations are life‐threatening at HA. Of AMS based on the assessment of oxygen saturation and sleep abnormalities is a matter of controversy (Naeije, 2010; Tannheimer et al., 2017).

A recent massive increase in tourism and sports activities at HA raises a need for the evaluation of adaptive cardiorespiratory variables, with adequate and portable devices (Ridolfi, Vetter, Solà, & Sartori, 2010). Photoplethysmography (PPG) associated with sleep apnea monitors and probes assessing respiration and PO_2_ have been widely used in this regard (Allen, 2007). Additionally, ambulatory blood pressure monitoring (ABPM) system has been used to investigate arterial BP at HA (Bilo et al., 2011). Indeed, altitude‐induced sleep disturbances have been widely reported and may negatively affect daytime performances, despite some uncertainty in specific adaptations due to age, gender, altitude working habit, living altitude, and altitude sickness (Bloch, Buenzli, Latshang, & Ulrich, 2015).

The purpose of this study was to investigate the cardiovascular and respiratory responses to HA hypobaric hypoxia, with attention to nocturnal adaptive response, in two mountaineers trekking high Himalayas: a white European (Italian) sojourner and a native Nepali Tamang. We also aimed to search for the plausible differences depending on ethnicity and chronic living conditions.

## 2 MATERIALS AND METHODS

The study was part of the medical research “Kanchenjunga Exploration & Physiology”, a subset of the broad project approved by the Ethics Review Board of the Nepal Health Research Council (NHRC) and performed under the ethical standards of the 1964 Helsinki declaration and all its amendments. All participants provided written informed consent to participate in the study.

**Figure 1:**
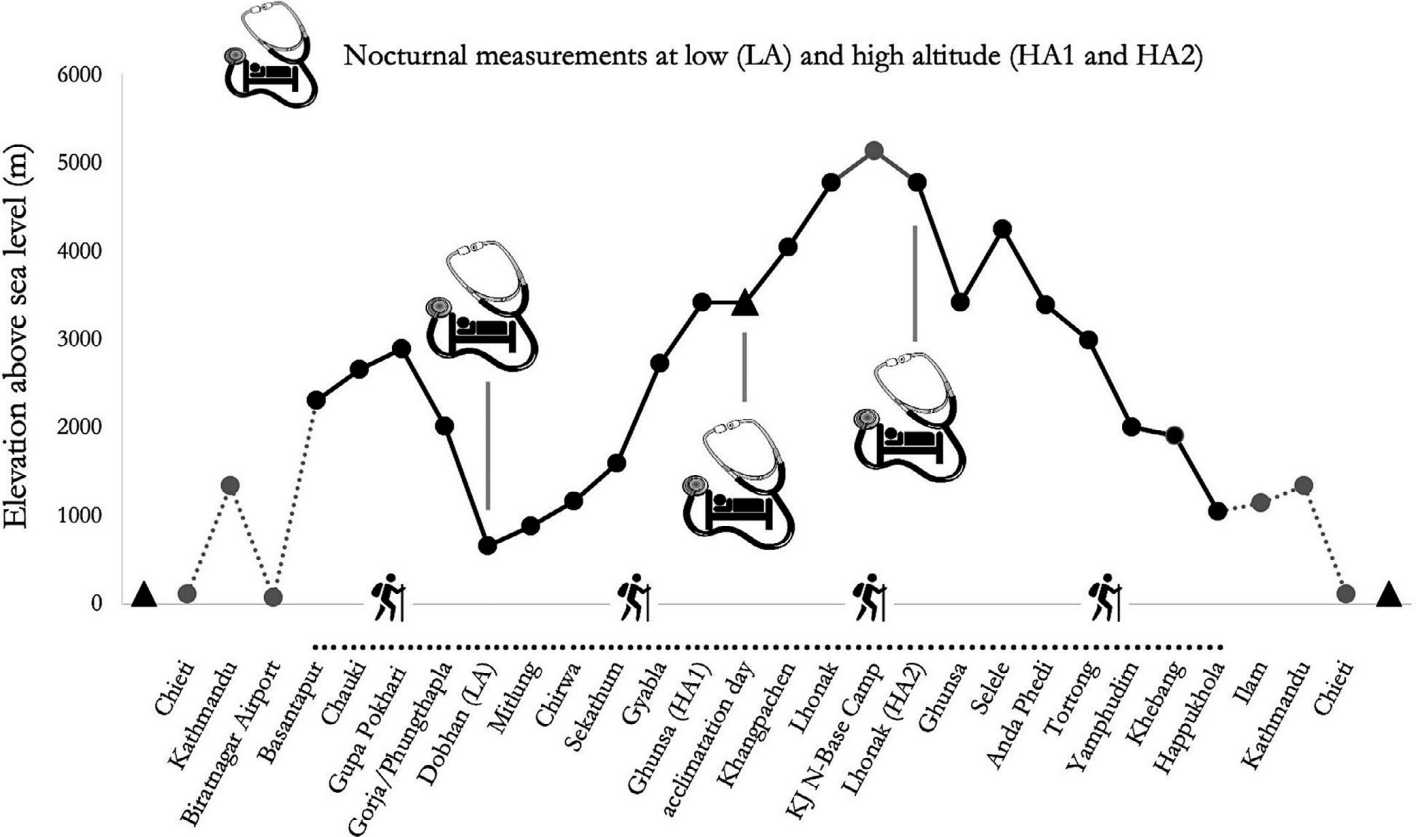
Altimetric scheme of “Kanchenjunga Exploration and Physiology”. Vertical lines denote three time points of assessment of nocturnal arterial blood pressure and oxygen saturation

The two participants completed a 19‐day trekking covering a circuit of 300 km over 16,000 m difference in altitude, with a daily average of 6 hr walking in the Nepali Himalayas. They underwent the assessment of nocturnal cardiovascular and breathing function, which was conducted in Dobhan (665 m), Ghunsa (3,427 m altitude), and Lhonak (4,780 m of altitude) (Figure 1). Both participants were considered well‐acclimated to altitude and neither suffered from altitude sickness. The Italian participant only took one acetazolamide pill of 250 mg daily, at 6 p.m. 2 days before reaching the highest altitude point.

**Figure 2:**
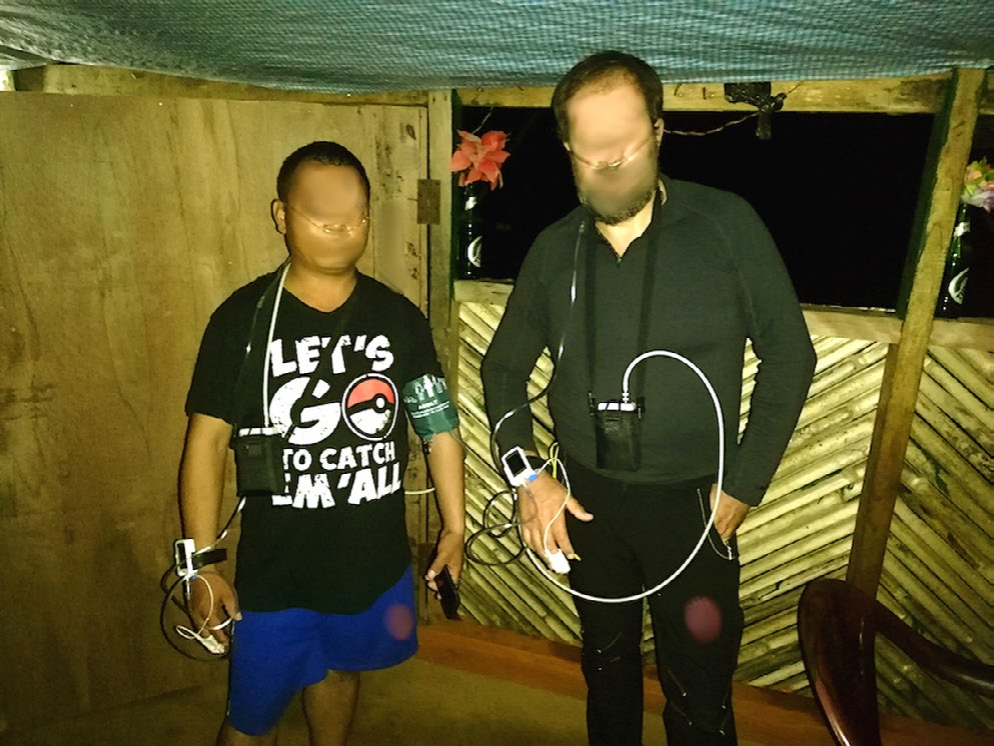
Tamang and Italian participants equipped with ABPM and sleep apnea monitor devices before sleeping

Characteristics of the two participants were as follows: ethnic white European (Italian trekker), nonsmoker, aged 48, body mass index (BMI) of 30.5 kg/m^2^; and ethnic Tamang (Nepali guide), light smoker, aged 40, BMI of 28.8 kg/m^2^. Neither participant reported any cardiovascular or respiratory disease, particularly heart arrhythmias or upper airway obstruction. Both participants wore two portable devices during sleep (Figure 2). The Italian trekker's abode normally was at sea level. However, he reported past HA experience more than 3 years before the current expedition. The Tamang guide also resided at low altitude, but reported frequent work‐related exposure to HA; on average, 3–5 expeditions *per* year.

Breathing and SpO_2_ were measured with two identical sleep apnea screening devices with 1 s intervals through a finger (WHO, 2011) and nasal probe (APN‐100; Contec Medical Systems Co. China). Mean arterial (MAP), systolic (SBP), diastolic blood pressure (DBP), and pulse pressure (PP) were measured automatically every 30 min with two identical oscillometric recorders (ABPM50; Contec Medical Systems Co. China). Nocturnal assessment took place between 22:00 and 6:00 o'clock. The average sleep duration was approximately 6–7 hr for both participants. We noted the time of going to sleep and waking time, along with changes in sleep behavior, urinary frequency (0–2 *per* night), and sleeping difficulty, such as delayed sleep onset or middle‐of‐the‐night insomnia.

Additionally, we assessed diurnal BP and SpO_2_ at daily basis in Kathmandu (1,450 m), before the expedition, and in Ghunsa and Lhonak, during the expedition. SBP and DBP data were taken in a sitting position at rest, with the cuff at heart level (Whelton et al., 2018), along with SpO_2_ and HR, in the morning before breakfast. Additionally, body weight and waist circumference were measured (World Health Organization, 2011). All the measurements were performed in duplicate.

Apnea was interpreted as a 80% decline in airflow or more of the baseline level lasting for 10 to 40 s (Thiriet, 2015). Breathing rate (BR) was normalized for the non‐apnea time, subtracting apnea time from the total time, and dividing the result by the number of breaths. From diurnal BP data, PP was calculated as a diastolic‐systolic difference, and MAP was calculated as SBP + 2DBP ÷ 3. Owing to the individual diaries of sleeping time, we controlled for any missing or disordered data caused by untoward changes in sleeping time or behavior. Any software‐related automatic removal of artifacts was manually rechecked.

## 3 RESULTS

Nighttime SBP and DBP increased during the ascent from 665 m to 3,427 m in both trekkers. During continuing ascent to 4,780 m, SBP remained at the increased level, while DBP further increased in the Tamang but not in the Italian. There was an increase in MAP during the ascent, which was also greater in the Tamang. PP remained about stable in the Tamang, while it decreased in the Italian at mid‐altitude, to increase even more at the highest altitude. HR continually increased from the lowest to highest altitude (Table 1), but the increase started from a lower value, being therefore relatively greater, in the Tamang. The percent of apneic episodes at sleep increased during the ascent in the Italian, who primarily showed such episodes also at low altitude, while it remained relatively low in Tamang. BR increased at the highest altitude in the Tamang, while it decreased in the Italian (Table 1).

**Table 1:** Nocturnal blood pressure (BP), heart rate (HR), breathing function, and oxygen saturation (SpO_2_) changes in high altitude trekkers

Trekker	Altitude	SBP^1^	DBP^2^	PP^3^	MAP^4^	HR^5^	SpO_2_^6^	Apnea time^7^	BR^8^	nBR^9^	HR^10^
Italian	665 m	115.7	70.1	45.7	81.6	57.2	95	13.0	13	15.0	56
	3,427 m	118.6	79.9	38.8	90.4	72.7	80	38.2	10	16.2	75
	4,780 m	128.7	77.8	50.9	96.1	65.7	84	44.7	7	12.4	62
Tamang	665 m	90.9	53.8	37.1	64.5	73.1	95	1.3	13	12.7	66
	3,427 m	103.8	67.8	36.0	78.0	72.9	89	0.7	17	17.3	77
	4,780 m	114.4	76.4	38.0	86.2	92.8	79	2.6	18	18.4	92

Note

Accuracy of the BP oscillometric recorder was as follows: HR 2 bpm, and BR 2 rpm. Interval between measurement was 30 min. Accuracy of the sleep apnea monitor was as follows: SpO2 2%, BR 2 rpm, and HR 2 bpm. Interval between measurements was 1 s.

^1^Systolic blood pressure (mmHg); ^2^Diastolic blood pressure (mmHg); ^3^Pulse pressure (mmHg); ^4^Mean arterial pressure (mmHg); ^5^Heart rate per min, measured by the BP oscillometric recorder; ^6^Peripheral oxygen saturation (%); ^7^(% of sleep time); ^8^Breaths per min; ^9^normalized breaths per min; ^10^Heart rate per min, measured by the sleep apnea monitor.

Diurnal SpO_2_ reflected a rebound after nocturnal reductions at all altitude levels. BR tended to be lower than that at night, and remained overall higher in the Tamang (Table 2). HR fluctuated at about the same level as that at nighttime. The increment in BP during diurnal ascent was primarily expressed by DBP and MAP values, while PP, differently from nocturnal data, was reduced at altitude in both trekkers. They both lost weight progressively with altitude. This reduction was more pronounced in the Italian, as he also had a clear reduction in waist circumference when compared with the Tamang.

**Table 2:** Diurnal blood pressure (BP) and oxygen saturation (SpO2) in high altitude trekkers. Accuracy of the devices was as follows: SpO2 2%, HR 2 bpm, BR 2 rpm, and BP 3 mmHg

Trekker	Altitude (m)	BMI^1^ (kg/m^2^)	WC^2^ (cm)	SpO_2_^3^* (%)	HR^4^* (bpm)	BR^5^ (bpm)	SBP^6^^ (mmHg)	DBP^7^^ (mmHg)	PP^8^ (mmHg)	MAP^9^ (mmHg)
Italian	1,450	30.6	110	97	64	14	136	82	54	100
	3,427	29.1	106	94	88	13	139	92	47	108
	4,780	28.7	103	92	86	10	137	94	43	108
Tamang	1,450	28.8	97	96	72	18	131	89	42	103
	3,427	28.3	96	93	76	10	124	94	30	104
	4,780	27.7	96	85	89	13	129	94	30	106

Note

Data are means of two measurements. ^1^Body mass index; ^2^Waist circumference; ^3^Peripheral oxygen saturation; ^4^Heart rate per min; ^5^Breaths per min; ^6^Systolic blood pressure; ^7^Diastolic blood pressure; ^8^Pulse pressure; ^9^Mean arterial pressure.

## 4 DISCUSSION

Sleep disturbances at altitude are common: in the systematic review of Bloch and colleagues (Bloch et al., 2015), the authors argued that subjective insomnia occurs at HA (i.e., 4,559 m) rather than at low (1,630 m) and moderate (2,590 m) altitudes, periodic breathing and arousals occur in lowlanders sojourning at altitude, and the effects of acclimatization on sleep are altitude dependent. However, sleep‐disordered breathing occur in a large proportion of highlanders, and a greater prevalence of sleep central apneas in highlanders compared with lowlanders has been reported (Pham et al., 2017), with a pathophysiological link to pulmonary hypertension (Latshang et al., 2017). So far, there is still uncertainty about the role of ethnicity and that of altitude working habit on cardiorespiratory nocturnal response depending on ethnicity and altitude working habit. In this scenario, this study is a comparative report of the nocturnal responses of the cardiorespiratory system to environmental hypobaric hypoxia in two ethnically different male Himalayas trekkers, a sojourner Italian and a native Nepali Tamang.

We found that oxygen saturation decreased in a way characteristic for HA sojourn in both participants (Baertschi, Dayhaw‐Barker, & Flammer, 2016). Arterial blood pressure showed a characteristic increase at HA (Narvaez‐Guerra, Herrera‐Enriquez, Medina‐Lezama, & Chirinos, 2018). The mean arterial pressure was the variable that showed the most expressive upward trends. We supplement current findings by showing day/night profile of BP changes and distinctly stronger increases at nighttime, which point to the potential peril of brain episodes developing during sleep in mountaineers. PP changes followed a diverse trend, ranging from 39 to 51 mmHg at nighttime in the Italian, while remained stable in the Tamang. In contrast, diurnal data rather showed a reduction in PP with altitude. If we consider the nocturnal data at the lowest and the highest point, HR and PP changes were unrelated. A larger increase in PP in Italian versus Tamang (11.4% vs. 2.4%, respectively) was in contrast to a larger increase in HR in Tamang versus Italian (26.9% vs. 14.9%, respectively). We can speculate that a larger fall in nocturnal SpO_2_ in Tamang versus Italian (−16.8% vs. −11.6%, respectively) might provoke a more intense compensatory chronotropic response. A reason may lie in changes of arterial stiffness (Said, Eppinga, Lipsic, Verweij, & van der Harst, 2018) caused by higher sympathetic activation with altitude.

The present study showed a nocturnal dipping of BP, with diurnal hypertension at altitude. Referring to nocturnal BP, although neither trekker had remarkable sleep disorders, the Italian took acetazolamide pills. Considering the time course of acetazolamide clearance (Van Berkel & Elefritz, 2018), it is rather doubtful that differential trends in BP changes between the Italian and Tamang trekkers could be due to the acetazolamide's diuretic effect.

We found that diurnal SpO_2_ was higher than nocturnal. However, the Italian had a lower SpO_2_ at moderate rather than HA. Considering the percentage of sleep‐related apneic episodes, which was increasing with altitude, there is a biological plausibility that an increase in periodic breathing, mostly consisting of central apneas, a characteristic feature of HA particularly in mountaineers aged over 40 (Said et al., 2018), could actually increase SpO_2_ during sleep, as an adaptive response. Even if we could not differentiate between peripheral obstructive and central apneic episodes, it is a reasonable assumption that we dealt central apneas. In this context, acetazolamide fails to prevent the increase in sleep apneas (Ainslie et al., 2013).

Breathing rate appeared not to be subject to adaptive responses at HA as the diurnal resting rate was unrelated to nocturnal rate. Of note, breathing rate of our trekkers was in a range characteristic for healthy adults, that is below 20 breaths/min (Cretikos et al., 2008). The Tamang trekker's breathing followed a typical response to hypoxia consisting of increased rate (San et al., 2013). In contrast, the Italian trekker's breathing rate, normalized for non‐apneic sleep, decreased at HA; a trend that would have been masked by a lack of data normalization. The pattern of sleep in the Italian, with the presence of apneic episodes at HA, a likely adaptive feature of sleep‐breathing function, deserves further exploratory research, particularly in the face of a report by Heinzer et al. showing the lack of breathing rate adaptation in response to hypobaric hypoxia (Heinzer et al., 2016). We suggest that a decrease in body weight that we noticed in the trekkers mass could affect the respiratory system, which entails an intertwined function (Homma & Masaoka, 2008; Littleton, 2012; Salome, King, & Berend, 2010; Thomas, Cowen, Hulands, & Milledge, 1989). We noticed an appreciable reduction in BMI and waist circumference in the Italian trekker as opposed to the Tamang. Therefore, in the context of typical respiratory adaptation, even in the absence of a specific mechanistic analysis, we suggest that a decrease in metabolism could reverse the hypoxia‐related adaptive augmentation of breathing rate.

The study has typical limitations of a field research in extreme conditions. We conclude that the cardiorespiratory function at extreme HA seems more affected during nighttime sleep, than during daytime physical activities related to ascent. That implies the plausibility of potentially life‐threatening brain episodes, such as HA cerebral edema, that could happen during nighttime rest and sleep at HA. Further studies should address the role of ethnicity, medications, and sociodemographic factors in the cardiorespiratory responses to hypobaric hypoxia.

## CONFLICT OF INTEREST

The authors declare no conflict of interest. The funders had no role in the design of the study; data collection, analyses, or interpretation of the data; writing of the manuscript; or in the decision to publish the results.

## AUTHORS CONTRIBUTIONS

Conceptualization, D.B. and V.V.; methodology, D.B. and V.V.; formal analysis, D.B. and V.V.; investigation, D.B. and V.V.; resources, V.V.; data curation, D.B. and V.V.; writing—original draft preparation, D.B., S.B., and V.V.; writing—review and editing, D.B., S.B., and V.V.; visualization, D.B., S.B., and V.V.; supervision, V.V.; project administration, V.V.; funding acquisition, V.V.
